# Effects of High Pressure Processing and Thermal Treatment on the Interaction between α-Lactalbumin and Pelargonium-3-Glucoside

**DOI:** 10.3390/molecules27154944

**Published:** 2022-08-03

**Authors:** Xuezhi Tian, Hui Zou, Peiqing Yang, Yan Ma, Yuwan Li, Liang Zhao, Yongtao Wang, Xiaojun Liao

**Affiliations:** 1 College of Food Science and Nutritional Engineering, China Agricultural University, Beijing 100083, China; txz@cau.edu.cn (X.T.); zouhui@sdau.edu.cn (H.Z.); ypq12138@cau.edu.cn (P.Y.); mayan1011314@sina.com (Y.M.); bs20203060505@cau.edu.cn (Y.L.); zhaoliang1987@cau.edu.cn (L.Z.); liaoxjun@cau.edu.cn (X.L.); 2 National Engineering Research Centre for Fruit and Vegetable Processing, Beijing 100083, China; 3 Key Lab of Fruit and Vegetable Processing, Ministry of Agriculture, Beijing 100083, China; 4 Beijing Key Laboratory for Food Nonthermal Processing, Beijing 100083, China; 5 Food Science and Engineering College, Shandong Agricultural University, Taian 271000, China; 6 Institute of Agro-Products Storage and Processing, Xinjiang Academy of Agricultural Sciences, Urumqi 830091, China

**Keywords:** high pressure processing, thermal treatment, α-Lactalbumin, pelargonidin-3-glucoside, binding behavior, interaction

## Abstract

In this study, high pressure processing (HPP) and thermal treatment were comparatively evaluated by examining their impacts on the binding behavior and interaction between α-lactalbumin (α-La) and pelargonium-3-glucoside (P3G) under pH values of 6.0, 7.4, and 8.0. The methods of circular dichroism spectroscopy, fluorescence quenching, dynamic light scattering, and molecular simulation were used to characterize the effects of processing-induced changes in protein structure, size distribution, binding site conformation, and residue charges on their binding characteristics between them. The results indicated that the thermal treatments significantly increased the quenching constants of the complex at pH 7.4/8.0 and 60/80 °C, as well as the accessible fraction of protein at pH 8.0/80 °C. Both HPP and thermal treatments increased the random coil content and showed limited effects on the α-helix and β-sheet contents of α-La and caused the aggregation of the complex to varying degrees. Molecular dynamic simulation and docking analyses revealed that the binding site of the complex did not change under different processing conditions, but the solvent-accessible surface area varied under different conditions.

## 1. Introduction

Anthocyanin is one of the most important phenol compounds in fruits and vegetables; it can generate an extensive range of colors [1] and has been explored as a functional food ingredient with the potential to prevent chronic diseases, such as cancer, inflammation, and diabetes [2,3,4]. Anthocyanin has a highly reactive structure with the delocalized π electronics, so its stability can be easily influenced by processing and storage conditions, such as pH; temperature; copigment structure and concentration; light; and oxygen, enzymes, proteins, and metallic ion contents [5].

A growing number of studies focusing on improving the stability of anthocyanin have been published in recent years; these studies have indicated that chemical modification [6,7], copigmentation interaction (the interaction of anthocyanin and other pigments and cofactors, such as polyphenols, organic acids, nucleotides, and metal ions [8]), and the interaction with food macromolecules, such as starch [9,10,11], polysaccharide [12,13] and protein [14,15,16], can all increase the stability of anthocyanin. Among these, the binding of protein and anthocyanin has been studied extensively due to their high safety and abundant sources, as well as precise available chemical structures and high-reliability application techniques [17]. Hydrogen bonds, hydrophobic interactions, ionic interactions, and van der Waals forces are the main driving forces contributing to this complex’s formation. Moreover, the changes in interactions between protein and anthocyanin can greatly influence the stability and functional performance of anthocyanin under different processing conditions (e.g., pressure, temperature, and pH) [18]. However, few studies have focused on the effect of processing conditions on the binding behavior and interactions of this complex.

High pressure processing (HPP) is a non-thermal food processing and preservation technology that causes minimal modification to the nutritional and sensory attributes of food products, only affecting the non-covalent bonds of biomacromolecules. During the last few decades, it has been widely used in the food industry to achieve desired effects on the texture, color, and flavor of foods [19]. Research focusing on the protein structure changes that take place under HPP has proven that proteins are penetrated by the solvent in the same way as chemical denaturants under HPP. The HPP leads to the population changes in conformation states that are more solvated than the native state by shifting the equilibrium toward conformations with newly exposed amino acid residues and decreasing the protein volume [20]. HPP has been proven to affect the interaction between protein and anthocyanin in the food matrix, but the mechanism of the effect has not been determined yet.

α-Lactalbumin is a globular protein derived from milk with a strong Ca^2+^ binding site and several distinct Zn^2+^ binding sites, which can bind Mg^2+^, Mn^2+^, Na^+^, and K^+^ as well. As a widely studied protein, α-La is a convenient tool for studying the interactions of a protein with other proteins, peptides, and low-molecular-weight phytochemicals of physiological significance [21,22,23]. Pelargonidin-3-glucoside is the component with the highest content of anthocyanin in strawberry, which contributes to its red color and antioxidant activity of strawberry [24]. Former research has reported the quenching parameters of α-La and pelargonidin-3-glucoside (P3G) under different high pressure conditions using fluorescence quenching and analyzed the protein structure and binding site via molecular dynamic simulations and molecular docking studies [22]. The results revealed that HPP increased the quenching reaction constants and the accessible fractions of α-La under the conditions of 100 MPa/pH 8.0 and 300 MPa/pH 8.0 [22]. However, the protein structure, size distribution, and their effects on the binding interactions under HPP were not studied.

In this work, the effects of HPP and thermal treatments on the binding constants and accessible fractions of P3G and α-La are compared, and the molecular mechanisms are elucidated by combining multispectral and computational simulations. These results could provide new insights into the impact of food processing conditions on anthocyanin stabilization strategies.

## 2. Materials and Methods

### 2.1. Materials

α-Lactalbumin (>85% with MW of 14.2 kDa) and pelargonidin-3-glucoside (chromatographic grade) were purchased from Sigma-Aldrich (Bornem, Belgium). The sodium dihydrogen phosphate and disodium hydrogen phosphate used for buffer solutions were of analytical grade and were purchased from Tianjin Chemical Factory (Tianjin, China). The deionized water used to prepare solutions was 18.2 MΩ and purified by an ultra-pure water system (Elga, High Wycombe, UK).

### 2.2. Preparation of Samples

The α-La solutions (5.0 × 10^−4^ mol/L) were prepared in phosphate buffers (50 mmol/L, pH 6.0, 7.4, and 8.0) and stirred for at least 1 h before use. The pelargonium-3-glucoside solution (2.0 × 10^−4^ mol/L) was prepared in hydrochloric acid solution (pH 2.0). Different volumes of anthocyanin, protein, and buffer solutions were transferred to small polyethylene bags to give a total volume of 0.5 mL; the final anthocyanin concentrations of 0, 1, 2, 4, 6, 8, and 10 × 10^−5^ mol/L; and the final protein concentration of 5.0 × 10^−5^ mol/L.

High pressure processing was conducted as follows: the polyethylene bags were sealed and processed using a 2.0 L unit (G7100 Food Lab, Stansted, UK). The pressure-transmitting fluid was a mixture of isopropyl alcohol and water with a volume ratio of 3:7, and the compression heating rate of this fluid was 3.3~3.5 °C/100 MPa. Samples were loaded into a high-pressure vessel, and then the vessel was sealed using a plug. The intensifier started to bump the pressure-transmitting fluid into the vessel to obtain the target treatments of 100 MPa/10 min, 300 MPa/10 min, and 500 MPa/10 min, respectively. The compression and decompression times were less than 3 min, and the temperature of the pressure-transmitting fluid was kept at 20 ± 3 °C.

Thermal treatments were conducted as follows: the reagent solutions were prepared and transferred into the polyethylene bags. Then, the polyethylene bags were sealed and kept in a water bath. The processing conditions were set as 40 °C/10 min, 60 °C/10 min, and 80 °C/10 min. Under the same processing conditions, the core temperature of the sample was directly detected with an infrared thermometer, and the time required to rise to the set temperature was determined through a series of preliminary experiments and not included in the holding time, and the samples treated at room temperature (20 ± 3 °C) under 0.1 MPa were used as blank control.

### 2.3. Fluorescence Measurement and Quenching Analysis

The fluorescence intensity of the solutions was determined with the TECAN spark 10M microplate reader (Tecan Group Ltd., Männedorf, Switzerland). The excitation and emission wavelength were set at 280 nm and 300~400 nm, with 1 nm intervals and 15 nm slit widths. The UV-Vis absorbance spectra of different anthocyanin concentrations were recorded with the TECAN spark 10M, and the wavelength range was set as 250~600 nm at 1 nm intervals. 

Following the treatment, 100 μL amounts of sample solutions were transferred to a 96-well plate, then the fluorescence spectra and UV-Vis absorbance spectra were scanned.

The fluorescence spectra were corrected by the following equation [25]:

(1) FC=Fm10(A1+A2)2

where F_c_ and F_m_ are the corrected and measured fluorescence intensities, and A_1_ and A_2_ are the anthocyanin absorbance at excitation and emission wavelengths.

The emission intensities at 350 nm were recorded, and the quenching rate constant and accessible fractions were calculated using the following equations:

(2) $$F/F_{0}=1+K_{\mathrm{sv}}\left[Q\right]$$

(3) $$F/{(F}_{0}\;-\;F)=1/f_{a}K_{a}\left[Q\right]+1/f_{a}$$

where F_0_ represents the fluorescence intensity without anthocyanin, F represents the fluorescence intensity at the given anthocyanin concentration, K_sv_ and K_a_ are the quenching constant and quenching constant of the accessible fraction, f_a_ is the fraction of the initial fluorescence that is accessible to the quencher, and [Q] is the concentration of anthocyanin.

### 2.4. Circular Dichroism Spectrometry

The secondary structures of α-La under HPP and thermal treatments were determined via the circular dichroism spectrometry of Chirascan plus (Applied Photophysics Ltd., Leatherhead, UK) [26]. The protein concentration was diluted to 0.1 mg/mL, the path length of the quartz cuvette was kept at 1 mm, and the light scan range was set as 180~260 nm.

### 2.5. Dynamic Light Scattering

The particle size distribution of α-La was analyzed using dynamic light scattering (Malvern Instrument, Malvern, UK) [27]. The intensity fluctuations in the scattered light were measured at an angle of 173° for 30 s, and the temperature was set at 25 ± 2 °C.

### 2.6. Molecular Dynamic Simulation

The structure of α-La was obtained from the Protein Data Bank, and the topology parameters were generated by the pdb2gmx module of GROMACS [28]. Molecular dynamic simulations were performed using the Amber99SB ILDN force field and TIP3P water model; then, the system energy was minimized by the conjugate gradient method. The system was equilibrated under NVT conditions for 1.0 ns with the position of protein-heavy atoms restrained by harmonic potentials. After this, another 10 ns of NVT simulation was conducted at 298 K and 1 atm, allowing all components of the system to move freely. Then, the system was equilibrated under the NPT condition for 20 ns. Finally, 30 ns of molecular dynamic simulations were carried out under the NPT condition and used to analyze the results. The equations of motion were integrated with a 2 fs time step, and no-bonded interactions were truncated with a 10 Å cutoff. For heating treatment, the system temperatures were set at 20 °C, 40 °C, 60 °C, and 80 °C, respectively, using the V-rescale. For HPP simulation, the system pressures were set at 0.1 MPa, 100 MPa, 300 MPa, and 500 MPa, respectively, using the Parrinello–Rahman algorithm. The structures and solvent-accessible surface areas of the proteins during the simulation period were extracted and analyzed for further study.

### 2.7. Molecular Docking

The α-La structures from the equilibrium stage of the molecular dynamic simulations were selected as the target ligand acceptor, and the binding sites were identified using the AutoDock software [29]. The rotatable bonds, active torsion of anthocyanin, and gasteiger charges of α-La were applied based on the default parameters. The grid box was designated to embody the entire protein, and the Lamarckian genetic algorithm was selected for the conformational search. The complexes with the minimum binding energies were extracted and used for pKa analysis, and the 2D interaction diagrams were drawn with Discovery Studio [30].

### 2.8. Residue pKa Analysis

The ionizable residues charges in the binding site of α-La in different pH conditions were identified by analyzing the residue pKa, which was calculated using the PROPKA software [31]. The structures extracted from different molecular dynamic simulations were uploaded to the official website in order to calculate the pKa of ionizable residues.

### 2.9. Statistical Analysis

In order to ensure the reliability of the experimental results, each measurement was carried out in triplicate. The SPSS 25.0 statistical software (SPSS Inc., Chicago, IL, USA) was used to perform an analysis of variance using one-way ANOVA, and significant differences were declared at *p* < 0.05.

## 3. Results and Discussion

### 3.1. Fluorescence Quenching Analysis

**Figure 1:**
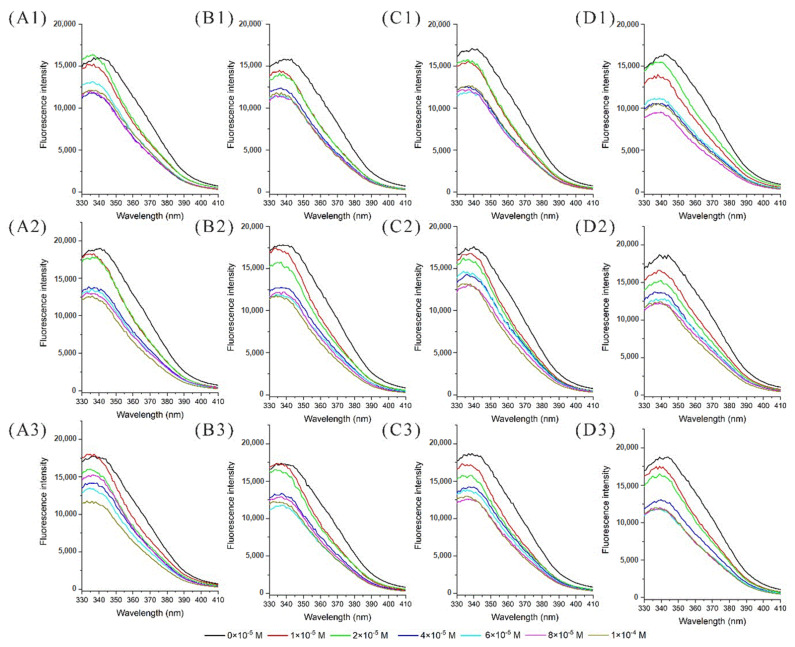
The fluorescence spectra of α-lactalbumin at different thermal treatments. (**A**–**D**): The fluorescence intensity of α-lactalbumin at 20 °C, 40 °C, 60 °C, and 80 °C, respectively; (**1**–**3**): the fluorescence intensity of α-lactalbumin at pH of 6.0, 7.4, and 8.0, respectively.

Fluorescence intensity and wavelength shift could provide information about tryptophan, tyrosine, and phenylalanine residues; the tryptophan residue was found to be dominant in absorbing the longest wavelength, displaying the largest extinction coefficient and being highly sensitive to the local environment [32]. The docking results obtained that the tryptophan residue in the binding site of α-La could be quenched by proton transfer from nearby anthocyanin [22]. In this experiment, the fluorescence spectra of α-La and P3G solutions subjected to different thermal treatments were recorded to examine the binding reaction. As shown in Figure 1, the fluorescence intensity of α-La was reduced with the increasing anthocyanin concentration due to the fluorescence quenching effect. Additionally, the maximum intensity wavelength at different thermal treatments changed little, indicating that the protein structures were not significantly affected by thermal treatments in the experimental conditions, and a similar result was found for HPP [22].

To examine the effects of thermal treatments on the quenching reactions of a-La and P3G, the Stern–Volmer equations were established by calculating the ratios of the fluorescence intensity in the absence and presence of anthocyanin (F_0_/F). The quenching constants and accessible fractions under the thermal treatments are shown in Table 1. The thermal treatments significantly increased the quenching constants at pH 7.4 and 8.0 with temperatures of 60 °C and 80 °C but did not affect the quenching constants in the solutions at pH 6.0. Moreover, the quenching constants obtained under the temperature of 80 °C were higher than the ones obtained at 20 °C, which might be related to the structure change under higher temperatures. The ANOVA results obtained for the accessible fractions showed a similar trend; the thermal treatments significantly increased the accessible fraction under the condition of pH 8.0 and 80 °C. Moreover, the accessible fractions at 80 °C were higher than the corresponding ones at 20 °C, and the comparatively larger errors obtained might have obscured the significant thermal effects.

The results of former research results indicated that HPP significantly increased the quenching constants and the accessible fractions of α-La [22], which is similar to the results obtained under the thermal treatments in this work. In order to clarify the mechanism of the two processing technologies affecting the binding behavior between α-La and P3G, the methods of circular dichroism spectrometry, dynamic light scattering, molecular dynamic simulation, and molecular docking were adopted to provide more information at the molecule level.

### 3.2. Protein Secondary Structure Analysis

Circular dichroism spectroscopy was applied to examine the effects of HPP and thermal treatments on the protein secondary structure in this experiment. The circular dichroism spectra of α-La under different treatments were scanned and processed by the CDNN software to calculate the secondary structure content (Figure S1). The processed data are shown in Table 2, where it can be seen that the α-helix content varied from 35.0% to 44.2% under different pH and pressure conditions and obviously increased with the pressure. The β-sheet content of HPP-treated samples showed few changes, but the random coil content decreased gradually with the increase in pressure under all pH conditions. These results indicated that HPP could promote the transformation of the α-helix of α-La to random coils, which represented the presence of unfolding conformation and might further induce the volume shrinkage of protein under pressure [22]. Due to its certain flexibility, the random coil structures are the main parts of the binding sites responsible for the recognition of small-molecule ligands [28,33], and in a few cases, the a-helix plays an important role [34]. For the thermally processed samples, the α-helix content decreased as the processing temperature increased to 80 °C but decreased sharply at 60 °C at all pH conditions. Simultaneously, the β-turn and random coil contents of the samples demonstrated opposite changes under the corresponding conditions. Thermal treatment exerted opposite effects on the secondary structure of samples compared with HPP, which revealed their different influence mechanisms. It has been recognized that HPP only affects the secondary bonds of proteins, especially hydrogen bonds and hydrophobic stacking, while thermal treatment can even cause the breakage of covalent bonds due to the high input energy [22]. Under the medium temperature treatment at 60 °C and slightly alkaline conditions (pH < 8.0), protein can be denatured, and even aggregation can be induced [35]. In conclusion, compared with HPP, the response of the secondary structure of α-La to thermal processing is more noticeable.

### 3.3. Size Distribution Analysis

As shown in Table 3, both HPP and thermal treatments could significantly affect the particle size distributions of α-La. The samples displayed two size distribution peaks at 229.40 nm and 3.95 nm under the condition of 0.1 MPa, while a new peak III appeared at around 40 nm in the samples treated at 100, 300, and 500 MPa. Meanwhile, the percentage of peak II intensity was reduced under pressure, and that of peak III intensity increased obviously instead. The peak at around 229 nm indicates that the protein mainly exists in the form of large polymers in solution, while the appearance of the new peak III demonstrates that the HPP might induce the formation of a compact conformational subset or small aggregates of the α-La monomer [34]. At pH 6.0, the intensity of peak I decreased under a pressure of 100 MPa but increased at 300 MPa and 500 MPa; similar phenomena also occurred in samples at pH values of 7.4 and 8.0. It seemed that the large aggregates of α-La were separated into two fractions with smaller particle sizes under 100 MPa, but the treatments of 300 MPa and 500 MPa essentially retained their original size and content of large particles. In the thermal treatments, no new peaks appeared in any of the samples. Compared with the control, the intensity of peak I of samples treated at 60 °C was significantly increased due to the aggregation. Moreover, the intensity of peak I significantly shifted from 227 nm to 870 nm, while peak II even disappeared under the condition of 80 °C/pH 8.0, which demonstrated the formation of large aggregates and even precipitates [35]. The change in particle size reflects the occurrence of protein unfolding or aggregation, which means that there were changes in the solvent accessibility of some amino acid residues, ultimately affecting the affinity and binding behavior with P3G.

The above results indicated that the HPP and thermal treatments could change the secondary structure and size distribution of α-La, which greatly influenced the binding interaction constants and accessible fractions between α-La and P3G. Therefore, molecular docking and molecular dynamic simulations were conducted to elucidate the effects of the above treatments on the α-La and P3G complex structure and residue charge at the binding site.

### 3.4. Analysis of Solvent Accessible Surface Area in Molecular Dynamic Simulation

The CD results indicated that HPP and thermal treatment changed the protein secondary structure, but the relationship between structural change and the binding interaction with P3G still needs to be explained. Molecular dynamic simulation is a suitable method with which to investigate the protein structure change and can provide insights into the molecular properties of protein on a different timescale [36]. In this experiment, the molecular dynamic simulations were processed to provide details on the protein conformations under different treatments and to help better understand the interactions occurring between α-La and P3G.

**Figure 2:**
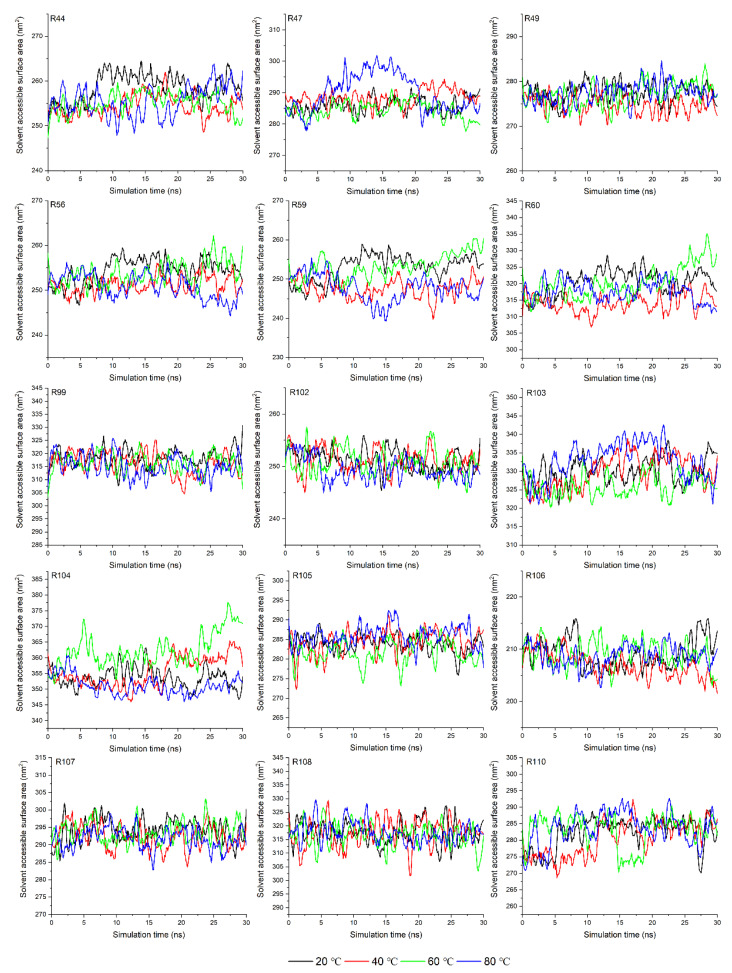
The solvent access area of α-lactalbumin residues in the binding site under different thermal treatments (letter R refers to the residues of α-Lactalbumin).

**Figure 3:**
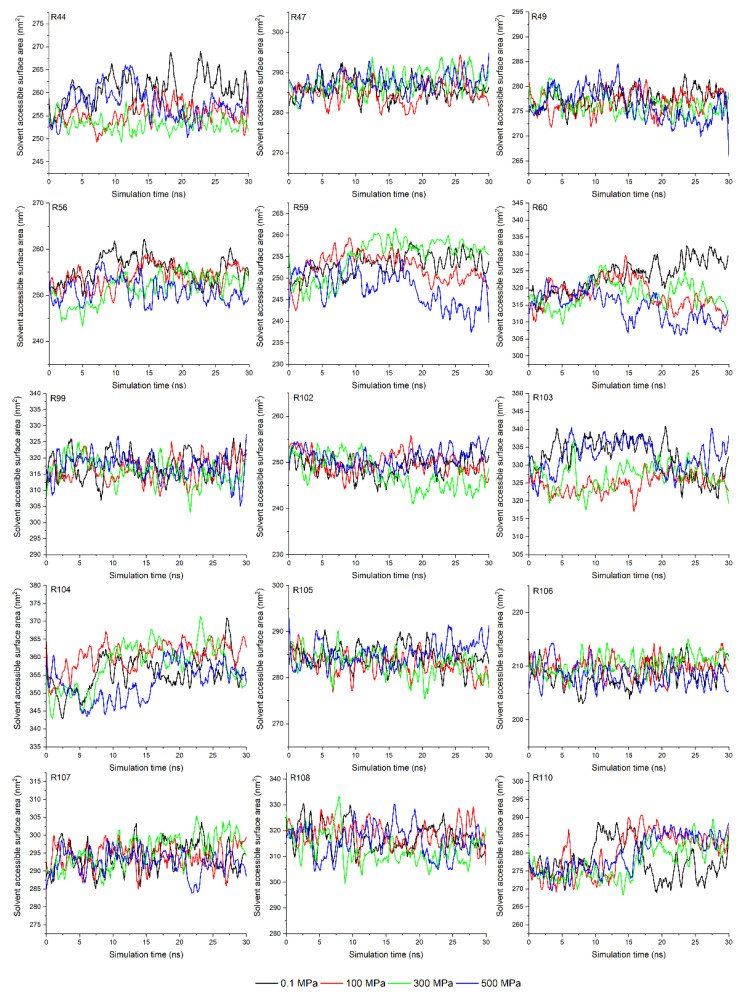
The solvent access area of α-lactalbumin residues in the binding site under different high pressure processing conditions (letter R refers to the residues of α-Lactalbumin).

The solvent-accessible surface area (SASA) of amino acid is a geometric measure of exposed environment proportion, which is related to the extent of the local environment, the solvent, and the protein core [37]. In this experiment, the SASA of amino acids in the binding site (determined by the molecular docking results shown in the following parts) were calculated with a 1.4 Å of probe diameter. As shown in Figure 2, the SASAs of the Asn44, Asn56, Asp59, and Phe60 residues were significantly reduced, while those of others were kept fundamentally stable despite the increase in temperature from 20 °C to 80 °C. The reduction in SASA might be related to the occurrence of aggregation, suggesting that the contact space between the α-La and P3G is reduced under the thermal treatment condition rather than cutting down the binding affinity between them [28]. The SASA of amino acids in the binding site under HPP showed a similar trend (Figure 3); more specifically, the SASA values of the Asn44, Asn56, Asp59, and Phe60 residues of α-La under HPP were smaller than the ones obtained at 0.1 MPa. This is also related to the occurrence of protein unfolding or aggregation under pressure [38,39]. Then, molecular docking was conducted to further analyze the relationship between amino acid structure change and binding interactions.

### 3.5. Molecular Docking Analysis

**Figure 4:**
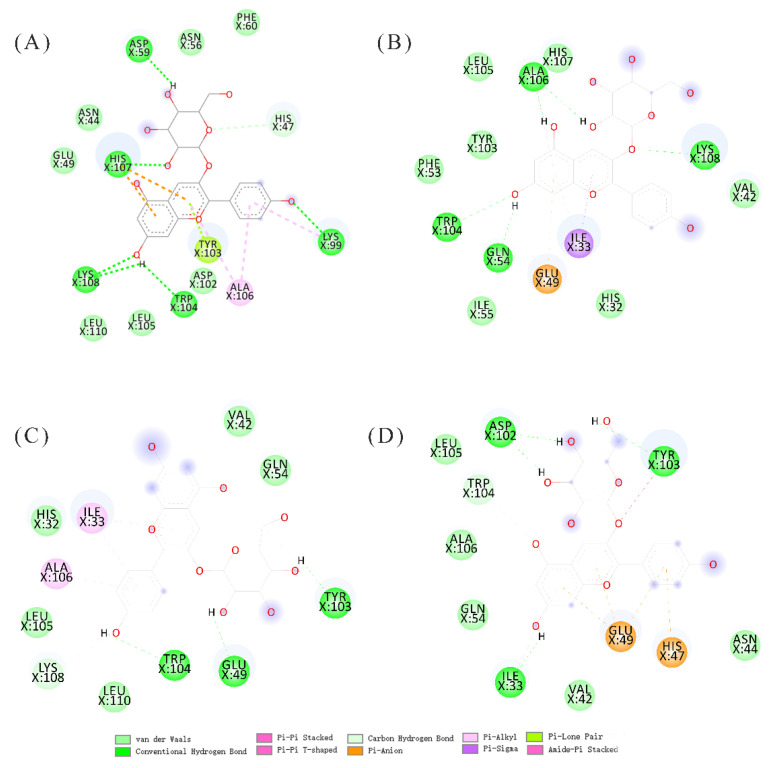
Molecular docking results of α-Lactalbumin and pelargonium-3-glucoside at different thermal treatments. (**A**–**D**): The interactions between residues of α-Lactalbumin and pelargonium-3-glucoside at 20 °C, 40 °C, 60 °C, and 80 °C, respectively.

The docking results indicated that the binding site of α-La was the typical Ca^2+^ site, and HHP did not change the binding site as reported by previous research [22]. The docking results of α-La and P3G under the different thermal treatments are shown in Figure 4. The binding sites with the lowest binding energy were the same at different conditions: the Ca^2+^ binding site. The binding structures indicated that high pressure processing and thermal treatment could not affect the binding behaviors of α-La and P3G, but the different structures might have led to the difference in binding energy. The binding energies obtained at 20 °C, 40 °C, 60 °C, and 80 °C were −8.53 kJ/mol, −6.0 kJ/mol, −5.45 kJ/mol, and −6.15 kJ/mol, respectively; the energy difference indicated that the binding models were different at different temperatures, and this was in accordance with the results obtained for the SASA. Moreover, the binding energies obtained at 100 MPa, 300 MPa, and 500 MPa were −5.12 kJ/mol, −5.58 kJ/mol, and −5.14 kJ/mol, respectively, which indicated that the complex of α-La and P3G treated with thermal treatment might have been more stable than the ones treated with HHP [22]. 

The hydrogen bonds, van der Waals forces, and hydrophobic stackings were the main driving forces for the model structures, which was in accordance with previous results [39,40,41]. Hydrogen bonds were found between P3G and Trp104 and Tyr103 residues of α-La in all conditions, which corroborated the previous results of fluorescence quenching. In addition, other residues were also found to establish van der Waals forces and hydrophobic stacking with P3G, such as Glu49, Asp59, Asp102, His107, and Lys108. These residues are ionizable, which suggests their ionization state changes with the pH of the solution and might thus affect the binding interactions between α-La and P3G. Therefore, the Propka software was used to evaluate the effects of pH on their binding interactions.

### 3.6. Residues pKa Analysis

Previous results have indicated that pH only affects the degree of binding and not the binding affinity for the interactions, with a lower pH leading to a stronger binding interaction since the dissociation of protein usually has more binding sites at a lower pH [40,42]. The charge of ionizable residues is determined by the desolvation penalty, back-bone and side-chain hydrogen bonds, and interactions with other charged groups, which cannot easily be calculated since the charge of ionizable residues can change for different pH and protein structures [31]. In this study, the protein structures at the equilibrium stage under different thermal treatments were selected as the initial structures with which to calculate the pKa of ionized resides using the propka 3.1. The pKa of all the ionized residues with the changed charges at the given pH are shown in Table 4.

The pKa of the HIS47 and HIS107 residues in the binding site varied from 6.32 to 7.47 and from 6.34 to 6.60 with the rise in temperature, respectively. When the solution pH increased from 6.0 to 8.0, the histidine residues could load the negative charge gradually. This changed charge also affected the interactions of α-La and P3G and caused the observed difference in quenching constants. For the HHP treated samples, the solution pH showed the same effects with different pKa values of HIS [22]. These effects indicated that a higher pH value changed the charge of negative residues and might have led to the binding interactions taking place with a higher hydrogen bond energy.

## 4. Conclusions

In this study, the effects of HPP and thermal treatment on the binding behavior of α-La and P3G were investigated, and the mechanism by which changes in secondary structure, particle size distribution, and residue charges under different processing conditions affect the binding interaction was elucidated. The results of fluorescence experiments indicated that both HPP and thermal treatment could affect the quenching constants and accessible fractions between α-La and P3G. Meanwhile, both two processing methods caused the transformation of a-helix to the random coil, and thermal treatments were shown to have more intense impacts. The particle size distribution analysis depicted that thermal treatment resulted in the severe aggregation and even precipitation of the α-La, while HPP tended to induce the appearance of a subpopulation of compact small aggregates with diameters of around 40 nm. Likewise, both treatments resulted in slight decreases in the SASA of some vital amino acid residues at the binding site, and the docking results indicated that the thermally processed α-La appeared to exhibit higher affinities for P3G. The hydrogen bonds, van der Waals forces, and hydrophobic stacking were the main driving forces for the binding of α-La and P3G, while changes in environmental pH could also cause changes in the ionization state of charged amino acid residues at the binding site, which in turn affected their binding interactions. In summary, the HPP and thermal treatments could cause changes in the secondary structure, particle size distribution, and SASA of α-La under different pH conditions, which ultimately contributed to differences in the binding behaviors between α-La and P3G.

## Figures and Tables

**Table 1:** The quenching constants and accessible fractions at different thermal treatments.

	pH	Control	40 °C	60 °C	80 °C
K_a_	6.0	0.498 ± 0.030 ^a^	0.388 ± 0.078 ^a^	0.495 ± 0.084 ^a^	0.542 ± 0.177 ^a^
	7.4	0.463 ± 0.042 ^a^	0.448 ± 0.092 ^ab^	0.304 ± 0.084 ^ab^	0.442 ± 0.075 ^b^
	8.0	0.392 ± 0.084 ^a^	0.462 ± 0.092 ^ab^	0.526 ± 0.015 ^bc^	0.633 ± 0.001 ^c^
f_a_	6.0	0.386 ± 0.194 ^a^	0.565 ± 0.237 ^a^	0.331 ± 0.054 ^a^	0.714 ± 0.371 ^a^
	7.4	0.417 ± 0.048 ^a^	0.515 ± 0.125 ^a^	0.464 ± 0.245 ^a^	0.473 ± 0.293 ^a^
	8.0	0.428 ± 0.244 ^a^	0.732 ± 0.272 ^ab^	0.563 ± 0.232 ^ab^	0.768 ± 0.196 ^b^

The numbers in quenching constants rows were shown as scientific notation (10^4^ mol/L). The different lower-case letters of the same row indicate significant difference existed during thermal treatments (*p* < 0.05).

**Table 2:** The second structure content of α-lactalbumin at different treatments.

pH	Content (%)	Control	100 MPa	300 MPa	500 MPa	40 °C	60 °C	80 °C
6.0	α-helix	37.60	37.80	38.70	44.20	45.60	30.10	44.30
	β-turn	17.00	17.10	17.00	18.00	16.50	19.20	16.40
	random coil	22.50	22.30	21.20	10.80	14.40	23.40	16.20
7.4	α-helix	38.20	40.70	40.70	40.50	43.90	32.30	47.90
	β-turn	18.50	17.90	17.90	17.90	16.10	18.60	15.80
	random coil	14.40	14.30	14.50	14.80	18.10	22.80	14.80
8.0	α-helix	35.00	38.60	39.60	41.30	43.40	42.80	41.30
	β-turn	17.90	17.30	17.70	17.80	16.10	16.60	17.10
	random coil	21.60	19.50	16.40	14.10	18.70	17.10	16.90

**Table 3:** The particle size distribution of α-lactalbumin under different treatments.

Treatments		Peak Intensity (nm)			Peak Intensity Percentage (%)		
		Peak I	Peak II	Peak III	Peak I	Peak II	Peak III
High pressure processing	Control/pH 6.0	229.40	3.95	0	56.53	43.46	0
	Control/pH 7.4	220.23	3.77	0	55.50	44.50	0
	Control/pH 8.0	191.54	3.83	0	57.06	42.94	0
	100 MPa/pH 6.0	189.56	4.06	39.78	51.43	30.93	17.66
	100 MPa/pH 7.4	178.32	3.80	23.40	55.90	29.38	14.74
	100 MPa/pH 8.0	136.03	3.55	13.73	61.13	25.20	13.67
	300 MPa/pH 6.0	247.53	4.09	49.99	49.63	31.16	19.20
	300 MPa/pH 7.4	297.65	4.00	46.87	47.70	31.83	19.32
	300 MPa/pH 8.0	126.25	3.85	17.17	54.80	34.95	10.20
	500 MPa/pH 6.0	187.32	3.97	33.37	54.14	35.98	9.92
	500 MPa/pH 7.4	225.13	10.30	28.84	54.65	26.23	19.10
	500 MPa/pH 8.0	261.16	3.89	30.44	59.80	25.50	14.70
Thermal treatment	40 °C/pH 6.0	197.26	3.76	0	45.63	54.36	0
	40 °C/pH 7.4	146.85	4.17	0	58.90	41.10	0
	40 °C/pH 8.0	140.10	3.67	0	47.05	52.95	0
	60 °C/pH 6.0	323.76	4.01	0	56.22	43.78	0
	60 °C/pH 7.4	320.95	3.97	0	59.32	40.68	0
	60 °C/pH 8.0	265.17	3.70	0	63.05	36.95	0
	80 °C/pH 6.0	188.40	3.73	0	61.00	39.00	0
	80 °C/pH 7.4	262.01	3.98	0	45.83	54.18	0
	80 °C/pH 8.0	870.6	0	0	100.00	0	0

**Table 4:** The pKa of histidine residues of α-lactalbumin at different thermal treatments.

Histidine Number	Control	40 °C	60 °C	80 °C
10	6.51	6.42	6.45	6.53
32	6.50	6.66	6.78	6.86
47	7.24	6.32	6.80	7.47
107	6.60	6.39	6.55	6.34

## Data Availability

The authors do not have permission to share data.

## Supplementary Materials

The following supporting information can be downloaded at: https://www.mdpi.com/article/10.3390/molecules27154944/s1, Figure S1: The circular dichroism spectroscopy of α-Lactalbumin at different treatments ((A–C): The circular dichroism spectroscopy of α-Lactalbumin at pH of 6.0, 7.4 and 8.0, respectively; 1 and 2 referred the thermal treatment and high pressure processing, respectively).
