# Supplementary material for: Effects of High Pressure Processing and Thermal Treatment on the Interaction between α-Lactalbumin and Pelargonium-3-Glucoside
Source: Molecules. 2022 Aug 3;27(15):4944. doi: 10.3390/molecules27154944 (PMC9370543; doi:10.3390/molecules27154944)
Supplement: Supplementary file 1 [file molecules-27-04944-s001.zip › molecules-1810648-supplementary.pdf]

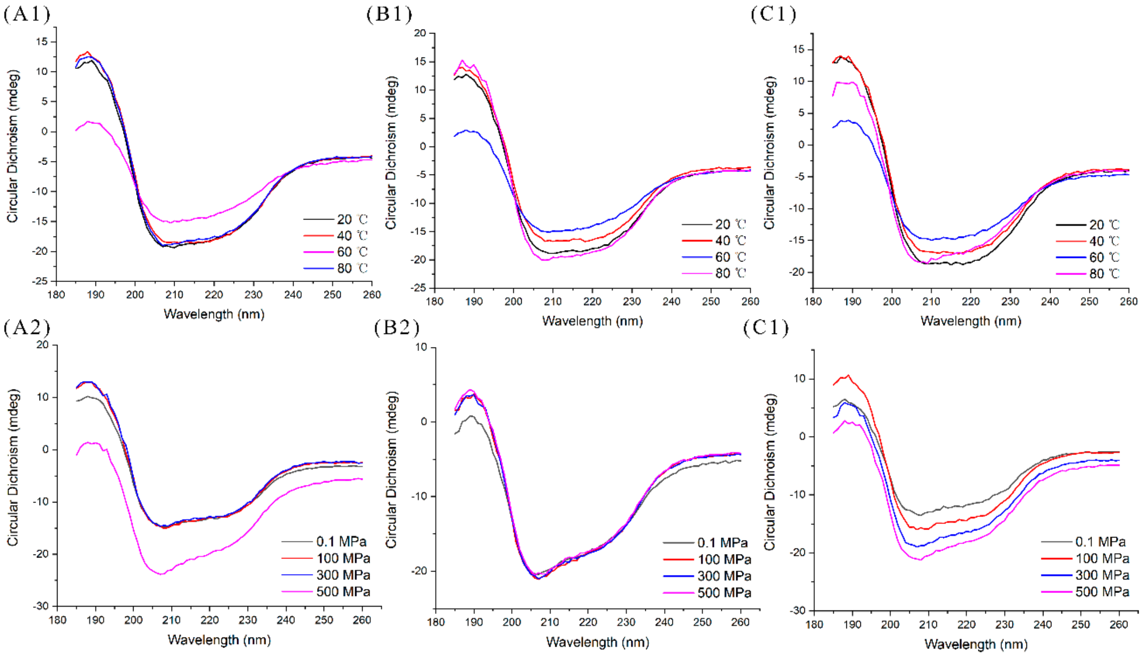

Figure S1. The circular dichroism spectroscopy of α-Lactalbumin at different treatments (A-C: The circular dichroism spectroscopy of α-Lactalbumin at pH of 6.0, 7.4 and 8.0, respectively; 1 and 2 referred the thermal treatment and high pressure processing, respectively.)
